# Optimal Compact Network for Micro-Expression Analysis System

**DOI:** 10.3390/s22114011

**Published:** 2022-05-25

**Authors:** Koo Sie-Min, Mohd Asyraf Zulkifley, Nor Azwan Mohamed Kamari

**Affiliations:** Department of Electrical, Electronic and Systems Engineering, Faculty of Engineering and Built Environment, Universiti Kebangsaan Malaysia, Bangi 43600, Malaysia; p109941@siswa.ukm.edu.my (K.S.-M.); azwank@ukm.edu.my (N.A.M.K.)

**Keywords:** micro-expression analysis, convolutional neural network, compact network, emotion classification

## Abstract

Micro-expression analysis is the study of subtle and fleeting facial expressions that convey genuine human emotions. Since such expressions cannot be controlled, many believe that it is an excellent way to reveal a human’s inner thoughts. Analyzing micro-expressions manually is a very time-consuming and complicated task, hence many researchers have incorporated deep learning techniques to produce a more efficient analysis system. However, the insufficient amount of micro-expression data has limited the network’s ability to be fully optimized, as overfitting is likely to occur if a deeper network is utilized. In this paper, a complete deep learning-based micro-expression analysis system is introduced that covers the two main components of a general automated system: spotting and recognition, with also an additional element of synthetic data augmentation. For the spotting part, an optimized continuous labeling scheme is introduced to spot the apex frame in a video. Once the apex frames have been recognized, they are passed to the generative adversarial network to produce an additional set of augmented apex frames. Meanwhile, for the recognition part, a novel convolutional neural network, coined as Optimal Compact Network (OC-Net), is introduced for the purpose of emotion recognition. The proposed system achieved the best F1-score of 0.69 in categorizing the emotions with the highest accuracy of 79.14%. In addition, the generated synthetic data used in the training phase also contributed to performance improvement of at least 0.61% for all tested networks. Therefore, the proposed optimized and compact deep learning system is suitable for mobile-based micro-expression analysis to detect the genuine human emotions.

## 1. Introduction

The recognition of facial expression is a basic function of the human brain [1]. Macro-expressions are intuitive reflections of human emotions, while micro-expressions (MEs) are more spontaneous expressions that are difficult to suppress or disguise. Analysis of MEs can therefore reveal genuine emotions, which is very useful in many sectors. Álvarez-Pato et al. in [2] showed that analyzing human emotions effectively can be used to predict consumer response. In addition, ME recognition has been employed in many fields, including lie detection, psychoanalysis, police interrogation, and national security [3]. However, this type of expression occurs in a very quick period of time (less than 0.5 seconds) and only involves very minuscule facial muscles, thus making the detection and analysis tasks very challenging [4].

**Figure 1:**
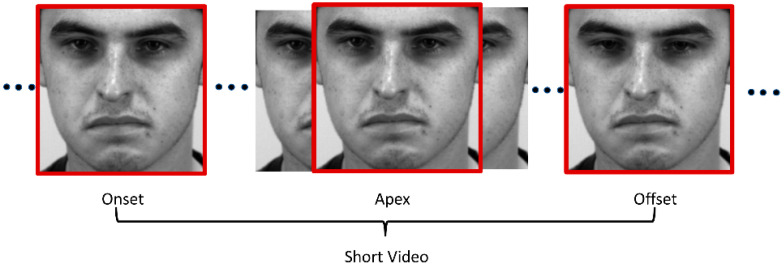
ME short video.

When a person tries to suppress their real emotions, they will try to maintain their neutral facial expression. However, the moment they fail to conceal their emotions, MEs will manifest. As a result, ME only occurs when the expression starts to appear from the neutral expression (onset), which is peaked at the apex frame and then reverts to the neutral expression again (offset) [5]. From a raw long ME video, a sliced sequence of onset-offset frames is defined as a “short video”, as visualized in Figure 1.

Apart from the beginning and ending frames that have special names, there is one frame between the onset and offset frames, called the apex frame, that contains the most expressive frame with maximum facial movement differences. Some researchers analyze only the apex frame for ME analysis, rather than the whole sequence of images, as this frame signifies the peak facial muscle movements, which is very informative for ME analysis [6]. Li et al. [7] showed that a single apex frame can provide sufficient information for an effective ME analysis system, and hence improving apex frame feature learning can significantly increase the deep model performance used for ME recognition. Owing to the importance of the apex frame, several studies [8,9,10,11,12] were dedicated to locate this most crucial frame accurately. However, the detection of the apex frame itself is very challenging due to the inconspicuous changes and the transient nature of ME.

Although the characteristics of MEs have made the development of this field intractable, their value has led to the continuous refinement of ME analysis techniques by researchers. The studies by Haggard et al. [13] and Ekman et al. [14] which detected and analyzed ME by slowing down the video playback are generally considered as the starting point for video-based ME research development. As the field has progressed, handcrafted ME features have been coupled with machine learning, as used in [6,15,16], followed by the current trend of using deep learning techniques. The main weakness of the conventional machine learning approach is the sub-optimal selection of the features [17], whereby the deep learning approach overcomes this limitation through optimized learning from a large amount of data. In fact, the application of deep learning technology has produced prominent results in several fields such as the trajectory control of robot manipulators [18], multiple hand gesture classification for teleoperated robot [19], electrocardiogram patterns monitoring [20] and automated ME analysis [21,22,23].

Yet, most studies tend to classify MEs without concern for effective ME spotting, whereby better ME spotting will lead to a more effective ME analysis [24]. Furthermore, a well-established automated ME analysis system based on deep learning is even less common. Therefore, in this paper, we have designed an optimized deep learning network to automatically analyze the MEs, involving both the spotting and recognition parts. However, the primary weakness of the existing methods can be traced to the lack of training data for optimal deep model fitting. Thus, this project proposes conditional generative adversarial networks (GAN) as a method to augment the training data through competing CNN networks between discriminator and generator networks. To further finetune the classification capability, the optimization of CNN networks has also been performed to produce better ME recognition.

As discussed above, the main contribution of this paper is the introduction of an optimized deep learning-based automated ME analysis system for both apex spotting and emotion classification modules. Firstly, we are the first to optimize the network and labeling process used in [12] for the apex spotting task. Secondly, we design a compact conditional DC-GAN to increase the amount of training data through synthetic optical flow images. Thirdly, we devise an optimized compact convolutional neural network (CNN), named Optimal Compact Network (OC-Net), to automatically classify the MEs into correct respective emotion categories, which produced the best classification performance compared to the state-of-the-art techniques. This paper is organized as follows: the related works are discussed in Section 2; while Section 3 details the materials and types of pre-processing features used, followed by the proposed methods and model architecture. Then, Section 4 reports and discusses the spotting and recognition performance. Finally, conclusions are given in Section 5 to highlight the advantages of the proposed network.

## 2. Related Works

### 2.1. Data Augmentation

The unique characteristics of ME as mentioned earlier, coupled with the complicated detection and analysis processes, make the task of ME dataset collection very arduous. The lack of ME data has therefore been seen as one of the major obstacles in the development of ME analysis. The studies in [25,26] also mentioned that a good deep learning model is heavily dependent on the availability of sufficient training data. However, collecting ME data that are useful and close enough to our natural responses is a very difficult task. Some challenges encountered during the ME acquisition process are stable lighting, high camera resolution, the difficulties of evoking accurate MEs, and the necessity for the researchers to determine whether the data are suitable to be included in the ME database [27]. These challenges will undoubtedly be a time-consuming process as well as very difficult, which is why, despite years of interest in ME analysis, the available ME databases have not expanded significantly. To the best of our knowledge, there are only three publicly available databases on spontaneous ME with good labeling information, SMIC [28], CASME II [3] and SAMM [29], which are popularly utilized nowadays for ME analysis. In total, there are at most 712 sets of MEs when these three databases are combined, which is relatively inadequate for optimal feature learning using deep learning models.

In 2014, the work in [30] introduced a simple generative adversarial network (GAN) for data augmentation purposes, which was subsequently popularly applied to numerous image analysis studies, such as CT denoising [31], lung cancer diagnosis [32], high-resolution skin lesion synthesis [33] and COVID-19 screening [34]. A GAN comprises two competing models; a generator that produces the desired output, and a discriminator which is responsible for distinguishing whether the data are real or fake. Among the many types of GANs, an interesting GAN architecture would be the conditional GAN. This type of GAN incorporates a conditional vector such as a one-hot encoded label [0 0 1] to represent different types of data, so that the conditional GAN can generate synthetic data based only on the chosen type [35]. Zulkifley et al. [34] utilized a conditional DC-GAN to equalize the imbalance dataset by synthesizing one class of the samples only and successfully improved the mean accuracy from 94.93% to 96.97% in detecting COVID-19. The remarkable performance achieved by GANs in the mentioned studies inspired us to leverage it in overcoming the problem of a lack of ME data.

### 2.2. Automated ME Analysis

**Figure 2:**
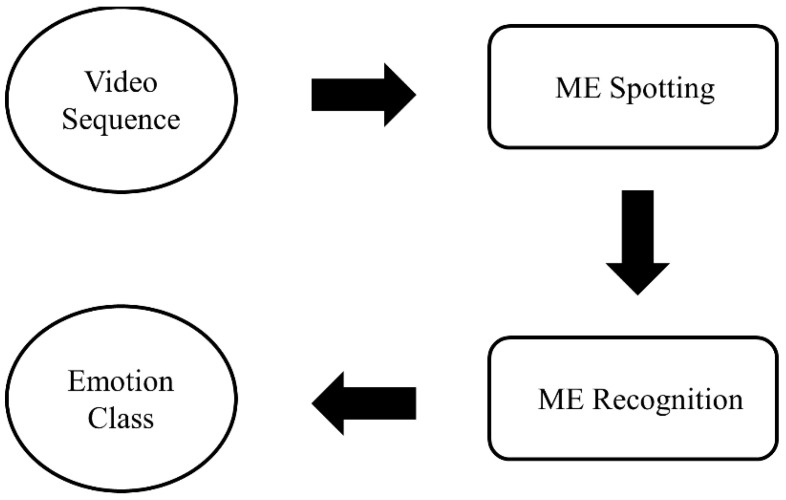
General flow of an automated micro expression analysis system.

An automated ME analysis can be broadly divided into two main parts: spotting and recognition tasks, as depicted in Figure 2. The former task involves detecting the apex frame in a short video of ME extracted from long video sequences, while the latter part involves classifying ME into respective emotion categories. The performance of an ME recognition system is highly dependent on the accuracy of ME spotting [24]. As mentioned in Section 1, some researchers prefer to spot the location of the apex frame instead of using the whole frame sequence in the short video.

The study in [12] employed the properties of a continuous function to infer the intensity of ME and consequently find the apex frame location using a sliding window of maximum label intensity (SW-Max) and maximum frame (Max) [11]. The best mean absolute error (MAE) of 11.36 frames was achieved when locating the apex frame tested on the CASME II dataset by employing an exponential continuous function label. However, on average, the authors showed that a linear continuous function performs better in terms of consistency and applicability. Then, the authors also further improved the concept in [36] with the aid of feedforward property networks. The short paths were inserted into the modified Visual Geometry Group Network (VGG-M) [37] using concatenation and summation properties. VGG-M is a network that is specifically design for mobile platform, which has been applied to various computer vision applications [38,39,40]. The study shows that networks with short paths concatenated outperform the original network and the feedforward networks with summation properties.

Based on one of the most recent publications on automated ME recognition systems that utilized the apex frame approach [21], the best deep learning algorithm produced less than 75% accuracy when tested with the CASME II dataset. The system used a convolutional neural network (CNN) architecture, named Off-ApexNet, fed with onset-apex optical flow features to classify human emotions. On the other hand, apex frame information was also used for micro-expression recognition in STSTNet [22]. In contrast to the two-dimensional optical flow used in Off-ApexNet, optical strain information was further added in STSTNet to result in a three-dimensional input.

## 3. Materials and Methods

### 3.1. Databases and Feature Extraction

**Figure 3:**
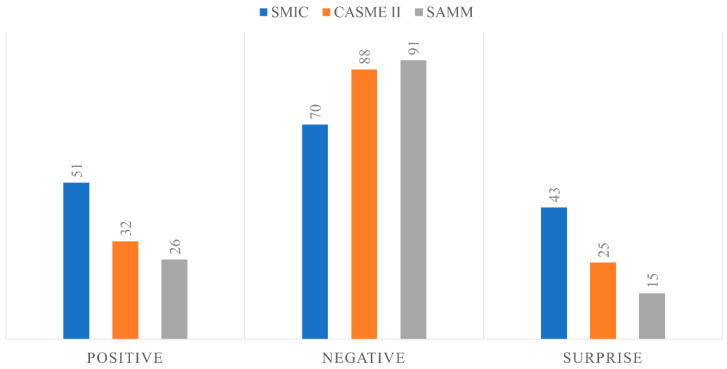
ME datasets.

Three public ME databases, SMIC [28], CASME II [3], and SAMM [29], were utilized in this study. These databases were chosen because they contain publicly available spontaneous ME data with annotated important frame information. For reference, only the SMIC database does not provide the apex frame information, but it does provide onset and offset frames. In CASME II, the emotions are categorized into 5 categories, while emotions in SAMM are divided into 7 classes, and SMIC has the lowest number of emotion categories at 3 classes. In order to utilize all three databases, this paper categorized the emotions into only 3 categories, namely positive, negative, and surprise. Negative emotion covered a wide range of emotions, including disgust, repression, contempt, fear, anger, and sadness, while positive emotion was derived from happiness, and the surprise class came only from surprise. Figure 3 summarizes the ME data of each database involved in this study.

**Figure 4:**
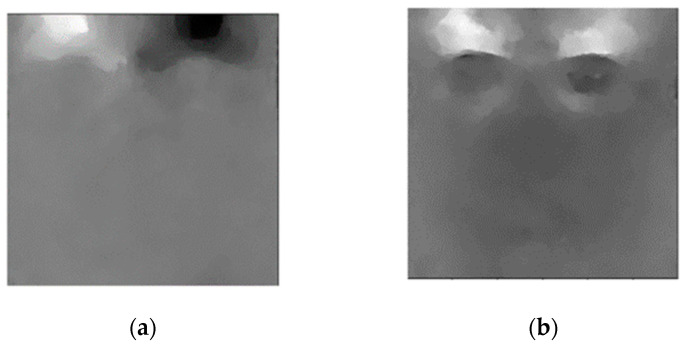
(**a**) Horizontal optical flow component; (**b**) vertical optical flow component.

The optical flow of MEs was extracted using the TV-L1 [41] method, which then became the input into the convolutional networks. For the apex spotting task, optical flows for the whole range of onset-offset frames were extracted, while for the ME recognition purpose, only optical flows of the apex frame were utilized. This type of flow feature represents the approximate subtle changes in facial muscle movements of the ME frames. Figure 4 shows samples of the horizontal and vertical optical flow of the apex frame, which were derived with respect to the onset frame.

### 3.2. Method Outline

**Figure 5:**
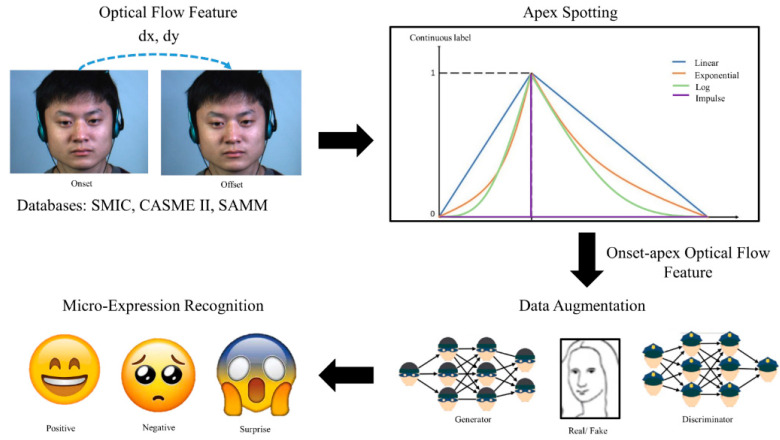
Workflow of the proposed method.

Figure 5 illustrates the general flow of the proposed method. ME optical flow features were fed into the CNNs for each of the three main steps: (1) apex spotting, (2) data augmentation, and (3) ME recognition.

### 3.3. Apex Spotting

**Figure 6:**
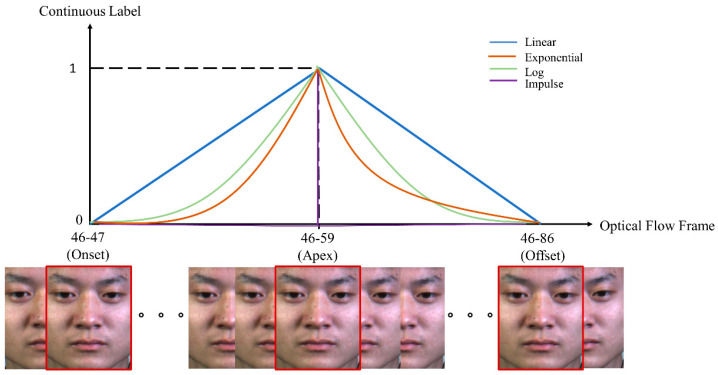
Continuous labeling scheme for the ME using optical flow input.

According to [12], the occurrence of ME followed the patterns of a continuous function. Therefore, continuing from this concept, we explored two new continuous functions to represent the labeling of the apex frame spotting, which were log and impulse functions. To be specific, we trained a CNN model to learn the relationship between optical flow features of onset-offset frames with respect to the continuous function labels. The continuous labels were pivoted on the onset, apex, and offset frames that were provided by the CASME II and SAMM databases. An example of ground truth labeling using a continuous function is illustrated in Figure 6. Then, the trained model was fed with the validation set optical flow, onset, and offset frames to generate the ME continuous label. Sliding windows with maximum (SW-Max) and maximum (Max) schemes were then used to further process the labels of each frame to locate the peak possibility of the apex frame.

**Figure 7:**
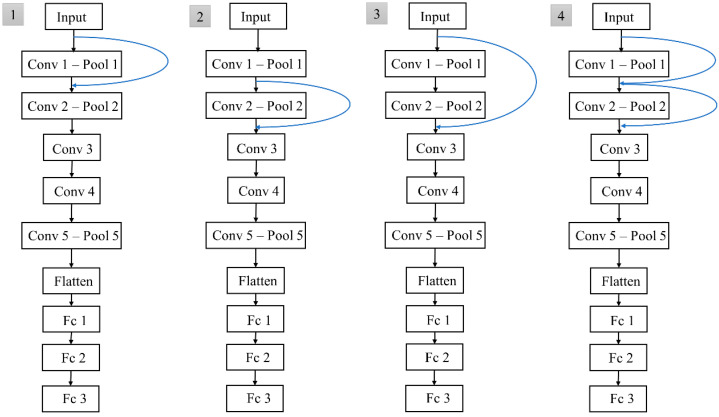
Four variations of the proposed feedforward networks. (**1**) Variation 1 uses a skip connection over a single layer applied to the first convolution layer input, (**2**) Variation 2 uses a skip connection over a single layer applied to the second convolution layer input, (**3**) Variation 3 uses a skip connection over two layers applied to the first convolution layer input, and (**4**) Variation 4 uses two skip connections over a single layer applied to the first and second convolution layer inputs.

The basic deep model used for apex frame spotting was the modified VGG-M network, whereby its architecture is summarized in Table 1. The model consisted of five convolution (Conv) layers, one flattened layer, and three fully connected (Fc) layers. The local response normalization (LRN) operator coupled with a maximum pooling (Max Pool) layer was then applied after the first two Conv layers. The activation function used for all Conv and Fc layers was the rectification linear Unit (ReLU), except for Fc 3, which used a soft-max function. Besides that, four variants of modified VGG-M networks with short paths were proposed, as shown in Figure 7. The continuous labeling scheme was generated using the basic models concatenated with these short paths, as used in [10], and was found to be able to predict the ME intensity changes very well. The purpose of this step was to determine the most effective continuous labeling function and the best CNN variant, which could then be used for apex frame spotting in the SMIC database.

### 3.4. Data Augmentation

**Figure 8:**
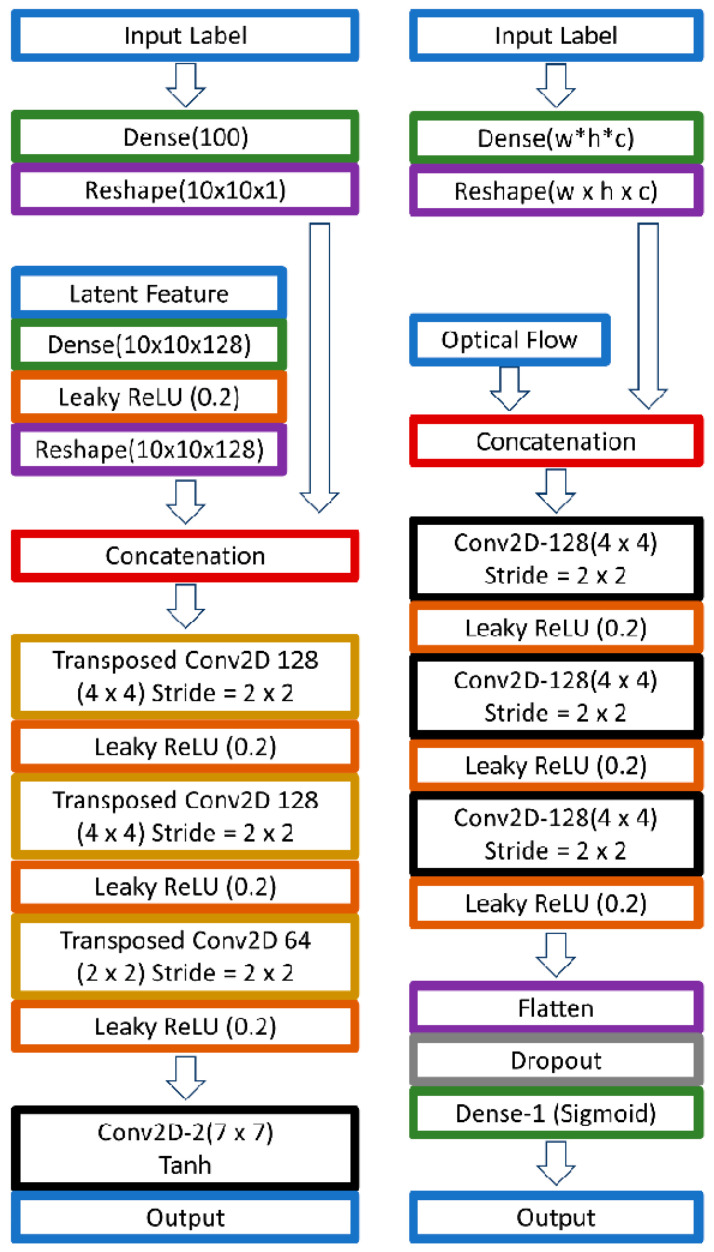
The proposed conditional Compact DC-GAN architecture that produces 80 × 80 synthetic onset-apex optical flow images. The network on the side is the architecture of the generator network, while the network on the right side is the architecture for the discriminator network.

Quality ME databases with detailed information are very limited, and thus in this study, synthetic datasets were generated to augment the existing data. Figure 8 illustrates the designed DC-GAN structure, which was used to generate ME onset-apex optical flow. Both the discriminator and generator used three-layer CNN, whereby the generator network employed transposed convolution to up-sample the feature maps, while the discriminator used strided convolution operators to down-sample the feature maps. The networks were trained with the onset-apex frame optical flow of the three databases. The apex frame for SMIC data was pre-determined using the best apex spotting method obtained in previous section.

We defined $X_{n}$ as the input *X* of layer *n* with a size of $w_{n}$*$h_{n}$*$c_{n}$, representing the width, height, and channel accordingly. The discriminator model D input, $X_{0}$, is equal to the concatenation of optical flow input, F, and the input label, L, as described in Equation (1). The input label L acts as the condition vectors, which represent the positive, negative and surprise data in one-hot-labels: [0 0 1], [0 1 0] and [1 0 0].

(1) $$X_{0}^{D}=F⊕L$$

The concatenation operation is represented by the symbol ⊕. Let C be the composite function for the convolution layer and leaky ReLu activation function, while R is the composite function of a drop out layer, a dense layer, and softmax activation function. Therefore, the proposed discriminator network D can be represented as:

(2) $$D=R^{D}\prod_{n=1}^{3}C_{n}^{D}X_{0}^{D}$$

On the other hand, the input of proposed generator G is denoted as $X_{0}^{G}$, which is the concatenation result of input label, L and latent feature, U.

(3) $$X_{0}^{G}=U⊕L$$

This input was fed into the proposed generator network G, which can be described in (4). Transposed convolution and leaky ReLu activation function are grouped as T, while $E^{G}$ indicates the output from a convolution layer and tanh activation function.

(4) G=U⊕L

The model training was performed with a learning rate = 0.002 and ADAM as the network optimizer with a 0.5 momentum parameter. The synthetic data generated were in the form of 80 × 80 pixels, which was sufficient for the subsequent ME analysis in this paper.

**Figure 9:**
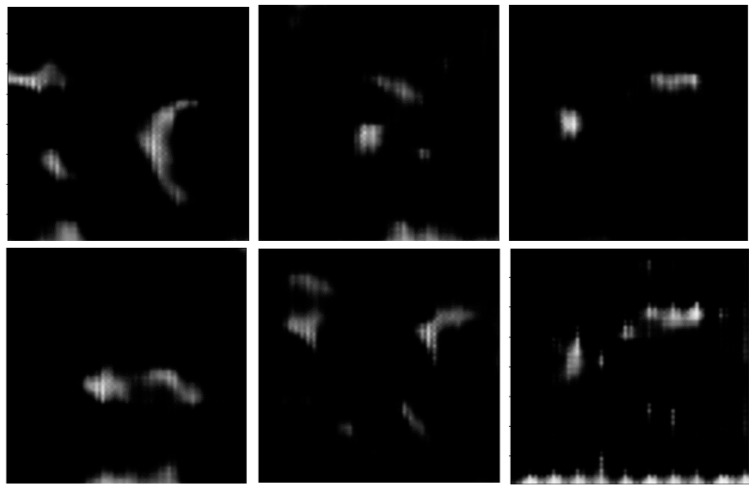
Samples of synthetically generated optical flow data.

Analysis of the GAN model’s performance was difficult due to the lack of an objective metric. An intuitive measure of performance could be executed by judging the samples’ visual quality [42]. However, it was difficult to gauge optical flow image quality, as shown in Figure 4. Therefore, the generated images were directly fed into the VGGM to augment the training set, and the training losses and accuracy were observed to determine whether the trained GAN model could generate good optical flow images or not. We also found that a deeper GAN model with more filters does not necessarily generate better outputs, as the generated outputs’ goal is to provide a better training dataset. Overfitting may occur if the training data are insufficient in number, especially for the case of fitting a deep learning model. Figure 9 depicts examples of synthetic ME optical flow generated by the proposed conditional DC-GAN.

### 3.5. ME Recognition

**Figure 10:**
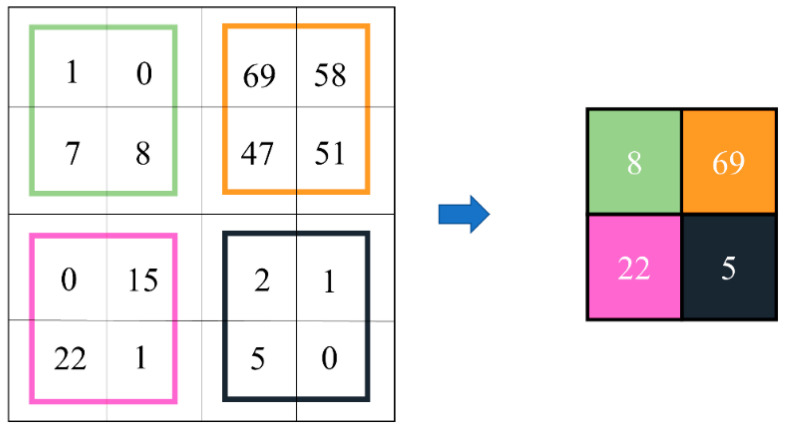
Maximum pooling operation for 4 × 4 feature map by 2 × 2 kernel with stride = 2.

The final step of ME analysis was to classify each ME into its respective emotion class. A compact optimized CNN model was designed to study the relationships between optical flow features of the onset-apex frames with the one-hot label. This labeling scheme followed the class representation as detailed in Section 3.4. After passing through a stack of convolutional and down-sampling layers, the model predicted the possibility of each tested video belonging to a particular emotion class. For example, if the model yielded an output of [0.7 0.1 0.2], the result indicated that the ME video belongs to a positive category. In this paper, the introduced OC-Net is a compact CNN model that comprises five convolution layers and three fully connected layers, which was optimized for the purpose of the ME recognition task. In designing the OC-Net, we emphasized retaining a large set of feature maps during the early layer so that lower-level features can be extracted optimally. We observed that reducing the feature map size during the early layer will cause a lot of important information loss, especially due to the maximum pooling operation, as shown in Figure 10. Although most of the feature information inside the orange color regions displayed high values, only the regional maximum value, which was 69, was extracted for further processing.

**Figure 11:**
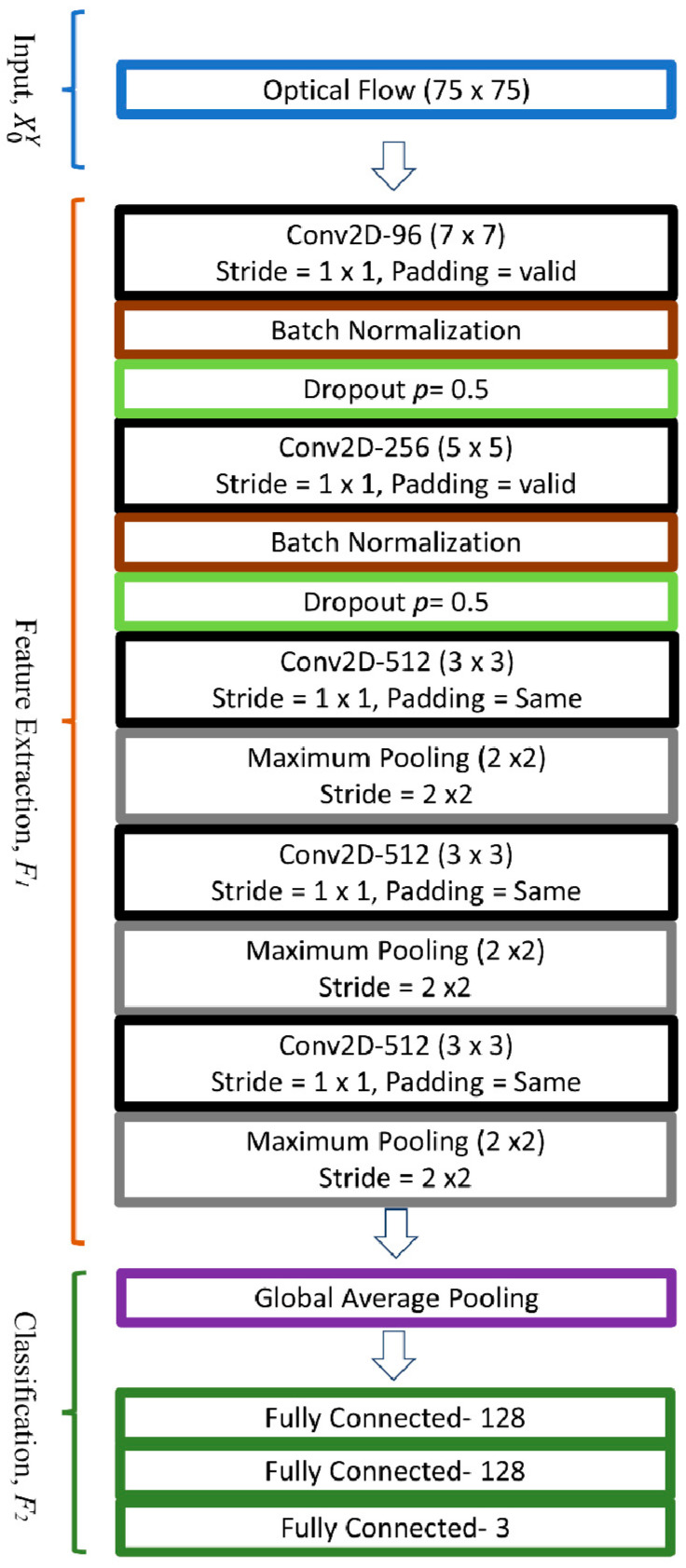
The proposed architecture of OC-Net that outputs 3 nodes of emotion class one-hot labels.

The complete structure of the OC-Net is shown in Figure 11. It is a simple compact CNN comprising three basic parts: input ($X_{0}^{Y}$), feature extraction ($F_{1}$) and classification ($F_{2}$). *Y* denotes the OC-Net classification network, thus:

(5) $$Y=F_{2}F_{1}X_{0}^{Y}$$

where the size of the input optical flow, $X_{0}^{Y}$, is 75 × 75. For the feature extraction stage, OC-Net consists of 5 units of convolution, $C_{n}$, where *n* is the number of *n*th layers. The convolution units, $C_{n},$ for *n* = 1, 2 consist of a convolution layer, batch normalization (BN), and a drop out layer, while for the remaining units, $C_{n},$ for *n* = 3, 4, 5 are composed of a convolution layer and a maximum pooling operator. All convolutional units use the ReLu activation function. The feature extraction, $F_{1},$ of *Y* can be expressed as:

(6) $$F_{1}=C_{5}C_{4}C_{3}C_{2}C_{1}X_{0}^{Y}$$

The down-pooling step was performed on *Y* using a maximum pooling operator in the last three convolution units to retain more feature information in $C_{1}$ and $C_{2}$. However, emphasizing larger feature maps in the early layers produces a heavier model, which can result in an overfitting problem and slows down the training process. Hence, BN was utilized to reduce the possibility of overfitting through the regularization process of the feature maps, which are usually inserted right after a convolutional layer [43]. Besides that, the addition of random dropout units was also explored to force the node’s connection to learn from various network settings, which would also reduce the possibility of overfitting problems. A dropout rate *p* = 0.5 was found to be the most suitable setting for the majority of the explored networks, which are also used in [44]. Besides that, dropout addition can also reduce the likelihood of variance shift issues.

The classification stage, $F_{2},$ included a global average pooling (GAP) [45] layer and three fully connected layers. The GAP helped to average out all the features and regularize the entire network structurally to prevent any overfitting, whereby the average pooling was only applied on the spatial dimension. For example, after a stack of convolution units, C_5 outputs (8, 8, 512) features were fed to GAP, and the resulting output was a 512-dimensional vector that was passed to a fully connected layer and softmax.

### 3.6. Performance Analysis

**Figure 12:**
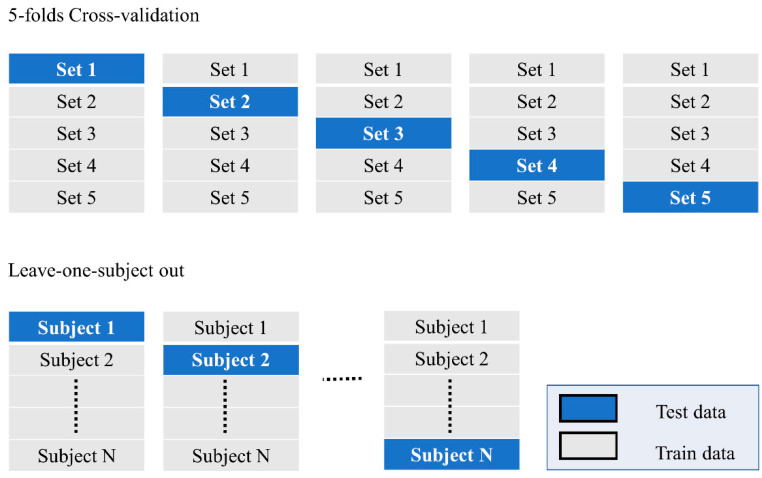
Cross-validation setting for the tested models.

The validation methods used for apex spotting and ME recognition tasks were different from each other. For the apex spotting task, 5-fold cross-validation was used, while a leave-one-subject-out cross-validation was employed in calculating the ME recognition task performance. Referring to Figure 12, performance analysis of the apex spotting task was executed by dividing the training and testing datasets according to the number of ME samples, which was performed to avoid bias that might occur due to the imbalanced training ME data between the various emotion classes. For the recognition task, the data were divided according to the number of subjects, so that bias among the subject could be avoided. Each of the subjects took turns to be the test data, while the rest became the training data, and this was repeated until all subjects had been tested.

After the cross-validation process, the effectiveness of apex frame spotting was measured using mean absolute error (MAE), while the effectiveness of ME classification into the respective emotion class was analyzed based on accuracy and F1-score. MAE represents the average frame error of the predicted apex frame with respect to the annotated ground truth apex frame, as computed in Equation (1), where the number of ME is represented by *T* and *d_i* is the frame difference between the *i*-th ground-truth and the predicted apex frame.

(7) $$\mathrm{MAE}=\frac{1}{T}\sum_{i}^{N}\left|d\_i\right|$$

The performance analysis of ME recognition task was based on the confusion matrix shown in Table 2. The accuracy metric is the most straightforward way of visualizing the reliability of ME recognition system, as shown in Equation (2), whereby it represents the proportion of correctly classified ME samples. However, for this study, due to the uneven class distribution, accuracy may not have been able to illustrate the ME recognition performance effectively, according to [46]. Thus, F1-score (Equation (5)) was also considered, whereby all the classes were equivalently important, regardless of the class size. Apart from that, two additional metrics of precision (Equation (3)) and recall (Equation (4)) were also employed to better capture the recognition performance of this study.

(8) $$\mathrm{Accuracy}=\frac{P+N}{P+N+\mathrm{FP}+\mathrm{FN}}$$

(9) $$\mathrm{Precision}=\frac{P}{P+\mathrm{FP}}$$

(10) $$\mathrm{Recall}=\frac{P}{P+\mathrm{FN}}$$

(11) $$F1\;\mathrm{score}=2\times \frac{\mathrm{Precision}\;\times \;\mathrm{Recall}}{\mathrm{Precision}+\mathrm{Recall}}$$

## 4. Results and Discussion

For the apex spotting task, CASME II and SAMM data are fed into the modified VGG-M model separately to analyze the spotting performance of various continuous labeling functions. The ME data are divided according to the ratio of 4:1 into respective training and testing sets, which then undergo a 5-fold cross-validation process. An ADAM optimizer with a total epoch = 1000, a batch size equal to 64 frames and a 0.0001 learning rate is set to train the models. Then, the likelihood of each tested frame being the apex frame of each ME video is predicted using the trained model. After the likelihood value of each frame is obtained, two schemes are utilized; the Max scheme is utilized to find the maximum probability frame, and the SW-Max scheme locates the sliding window (window size = 9) with the maximum probability as the apex frame, which means the neighboring frames’ probabilities are also considered during the decision-making process.

A previous study showed that a linear continuous function can cope well with the SW-Max scheme that performs better than the exponential function, with the best average MAE of 14.37 frames. Hereby, we investigate further the relationship between various continuous functions and the ME analysis performance through log and impulse functions. Table 3 shows the average error performance of these functions in locating the apex frames. From the table, we can see that the impulse function is not suitable for the apex spotting application as the errors are relatively large compared to the other functions. The maximum MAE value, which is the worst performance is achieved by impulse function with an error of 24.33 frames when locating the apex frame, tested on the SAMM dataset, while the performance of the proposed log function is stunning, especially when the ME log continuous label is further processed using the SW-Max scheme with a low error rate of only 10.36 frames, tested on the CASME II dataset. This improvement is even better compared to the best method in [12] with the lowest MAE value of 11.36 frames.

On average, the SW-Max scheme coupled with the log continuous label managed to locate the apex frames with only 12.44 frames of error. Furthermore, the SW-Max scheme generally outperforms the Max method according to the results tabulated in Table 3. The maximum MAE of the Max scheme is 24.33 frames when it is processed using impulse continuous labels based on the SAMM dataset, while the SW-Max scheme has a much lower maximum error of only 14.96 MAE. After analyzing Table 3, the results show that single frame information is less effective, whereby the neighboring frame information can smooth out the error in predicting the apex frame.

Apart from that, the feedforward property is also found to be able to improve the apex spotting performance, and hence, we concatenate the short path connections in Figure 6 to further analyze the performance of the log continuous function. Table 4 tabulates the MAE values of the model with feedforward properties in generating the log continuous label for apex frame spotting, tested on the CASME II dataset. The log continuous label generated by the feedforward network managed to represent the ME data better compared to the original VGG-M. The best MAE achieved is only 10.28 frames, but a linear continuous label with the aid of the feedforward networks by Koo et al. performs even better with the lowest MAE of 8.46 frames. Thus, a linear continuous function is the best overall function for labeling the apex frame network. Therefore, in this study, apex frames for the SMIC data are located using the network with a concatenated short path to the modified VGG-M coupled with the linear continuous labeling function.

For each ME emotion category, 100 synthetic optical flows are generated as the augmented data, resulting in a total of 300 synthetic data generated using the proposed conditional DC-GAN. These augmented data are only applicable during the training process, and not in the testing process. A leave-one-subject-out validation method is applied in analyzing the ME recognition task performance on CASME II, SAMM, and SMIC, which is tabulated in Table 5. In other words, the initial ME training data of 67 subjects are now augmented by 300 artificially generated data.

Modified VGG-M shown in Table 1 is also used as the benchmark for the ME recognition task, whereby the single output node is changed to three nodes. The overall ME recognition performance of the modified VGG-M model significantly improved from 72.34% accuracy and 58.50% F1-score to 78.3% accuracy and 67.57% F1-score when the training data are augmented using synthetic data. The model trained by a combination of real ME data and synthetic data managed to categorize the emotions better for both CASME II and SMIC datasets. Improvement of around 12.88% accuracy and 19.31% F1-score for the CASME II dataset can be observed on average, while for the SMIC dataset, the accuracy value improved by 8.13%, and the F1-score by 12.2%. On the other hand, model performance on the SAMM dataset decreased by 4.04% regarding accuracy and 6.06% regarding F1-score.

For the ME recognition task, the proposed OC-Net is trained using an ADAM optimizer with a learning rate of 0.00006 for 500 epochs with leave-one-subject-out cross-validation. The full performance results are tabulated in Table 6. Modified VGG-M is also used as the benchmark for ME recognition performance as both the modified VGG-M and OC-Net are compact CNN models with five convolution layers and three fully connected layers. When OC-Net performance is compared to the modified VGG-M, whereby no data augmentation scheme is applied, the accuracy and F1-score improved from 72.34% and 58.50% to 78.53% and 67.80%, respectively.

Table 7 shows the comparison between OC-Net variants in classifying ME emotion categories. The analysis is performed by adding dropout layers to the model layer one-by-one. The results indicate that the insertion of dropout layers *p* = 0.5 improved ME recognition performance from 65.31% to the best F1-score of 67.80%. OC-Net with larger feature maps in the early layer performs better than the modified VGG-M, which underwent down-sampled processes in the early stage. Even without the dropout layers, OC-Net still achieved an F1-score 6.81% better than that of the modified VGG-M. This performance improvement can be attributed to the deep model’s better ability in handling early layer feature loss due to the pooling operations. Besides that, OC-Net with dropout layers right after every BN layer also produced the best ME recognition performance.

**Figure 13:**
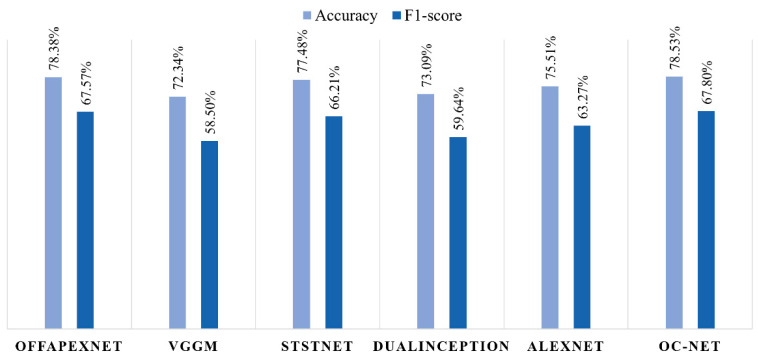
ME recognition performance of OC-Net and its benchmarked models.

When compared to the other state-of-the-art CNN models in micro-expression analysis, OC-Net still produces the best emotion recognition in terms of accuracy and F1-score. Only OffApexNet [25], with an accuracy of 78.38% and F1-score of 67.57%, came in a close second to OC-Net’s performance. STSTNet [26] recognized the ME with a performance of 77.48% accuracy and 66.21% F1-score, which is ranked in third place, followed by AlexNet [36], DualInception [27], and VGG-M [11]. STSTNet uses a unique set of inputs, which are fed with three-dimensional optical flow features: horizontal and vertical components of the optical flow and optical strain, while the other models use standardized inputs of two-dimensional optical flow features. Figure 13 illustrates the ME recognition performance of different state-of-the-art CNN models in micro-expression recognition systems.

Based on the good performance of data augmentation using GAN as shown in Table 5, the following experiments use synthetic optical flows, which are fed into OC-Net during the training phase. Table 8 shows that the model performance improved from 78.53% accuracy to 79.14%, while F1-score improved from 67.80% to 68.71%. Moreover, the impact of GAN is minimal for the CASME II dataset as the performance results remained the same for OC-Net ME recognition with and without GAN data. On the other hand, the ability of OC-Net in classifying the SAMM dataset also improved. It is also observable that OC-Net with synthetic optical flow performs better than the modified VGG-M.

Overall, the ME recognition results of the CASME II are much better than the other two databases. This is mainly because the micro-expressions used in the CASME II database were all taken from Chinese youth, which is in contrast with the SAMM database that utilized 28 subjects from 13 different ethnic groups. In fact, different ethnic groups have different typical appearances, both in terms of face shape and skull shape. Murray et al. [47] showed that face shape and face surface will surely affect facial expression representation. In addition, the SAMM database has the widest variety of negative expressions among the three tested databases with five types of expressions: contempt, disgust, fear, anger, and sadness. Although these types of expressions are the same in a sense, whereby all emotions are considered as negative emotions, the way they are expressed and manifested is totally different. Thus, it is hard to evoke the augmented data for the negative cases accurately, as the labeling scheme is only singular. In the case of the SMIC database, the performance of the SMIC database is greatly diminished by the absence of ground truth apex frame information. Although the apex spotting method we used produced only small errors on CASME II and SAMM, there is no performance guarantee of the extracted apex location in SMIC, as no ground truth is provided.

The proposed method only focuses on the fine-cropped facial area which perpendicularly faces the camera. Future research could include different angles of facial ME such as a slightly sideways head, which occurs frequently in real life, as humans are very mobile. Increasing ethnic diversity is also a notable future research direction to further increase the system’s robustness. Additionally, in this paper, we have mentioned that the wide range of negative expressions could be better sorted in future studies to produce a more balanced dataset between emotions. This would lead to better learning of each category by the deep learning models. Finally, more variants of DC-GAN can be implemented to generate more high-quality synthetic data in order to produce a more diverse training dataset for better model optimization.

## 5. Conclusions

In conclusion, this paper has managed to propose an ME analysis system that produced the best performance compared to the benchmarked state-of-the-art methods, with the best accuracy being 79.14%. Meanwhile, the log continuous labeling scheme, generated for the CASME II dataset, achieved the lowest MAE of 10.28 frames. Although both of the proposed continuous functions, log and impulse functions do not surpass the performance of the linear continuous function in spotting the apex frame, we are able to prove the pattern that ME occurs with gradually incremental and decremental changes in facial intensity. Moreover, we also show that a model that utilizes larger feature maps in the early layers performs better compared to the model that immediately applied down-pooling layers. Our synthetic ME optical flows also improved the recognition performance, whereby the modified VGG-M model improved from 58.50% to 67.57% in terms of F1-score, while OC-Net managed to improve from 67.80% to 68.71%. For future work, more variants of conditional DC-GAN will be explored for better ME analysis.

## Figures and Tables

**Table 1:** Modified VGG-M configuration.

Layer	Kernel Number	Kernel Size	Stride	Padding	Activation Function	Output Shape
Input	-	-	-	-	-	(75, 75, 2)
Conv 1	96	7 × 7	2 × 2	Valid	ReLU	(35, 35, 96)
LRN						
Max Pool 1	-	3 × 3	1 × 1	-	-	(17, 17, 96)
Conv 2	256	5 × 5	2 × 2	Valid	ReLU	(7, 7, 256)
LRN						
Max Pool 2	-	3 × 3	1 × 1	-	-	(3, 3, 256)
Conv 3	512	3 × 3	1 × 1	Same	ReLU	(3, 3, 512)
Conv 4	512	3 × 3	1 × 1	Same	ReLU	(3, 3, 512)
Conv 5	512	3 × 3	1 × 1	Same	ReLU	(3, 3, 512)
Max Pool 5	-	3 × 3	2 × 2	-	-	(1, 1, 512)
Flatten	-	-		-	-	(512)
Fc 1	128	-		-	ReLU	(128)
Fc 2	128	-		-	ReLU	(128)
Fc 3	1	-		-	Softmax	(1)

**Table 2:** Confusion matrix used in performance analysis.

	Actual		
		Positive	Negative
Predicted	Positive	True Positive, P	False Positive, FP
	Negative	False Negative, FN	True Negative, N

**Table 3:** MAE of Apex spotting using various continuous label functions.

Continuous Function	CASME II		SAMM	
	Max	SW-Max	Max	SW-Max
Log	11.16	10.36	14.96	14.52
Impulse	21.46	21.00	24.33	17.26

**Table 4:** MAE of apex spotting using log function and concatenation properties.

Log	1	2	3	4
Max	11.52	12.06	10.58	11.52
SW-Max	10.28	11.72	10.54	10.82

**Table 5:** ME recognition performance of modified VGGM with and without augmented training data.

Database	Without GAN		With GAN	
	Accuracy (%)	F1-Score (%)	Accuracy (%)	F1-Score (%)
Overall	72.34	58.50	78.38	67.57
CASME II	76.09	64.14	88.97	83.45
SAMM	78.79	68.18	74.75	62.12
SMIC	63.82	45.73	71.95	57.93

**Table 6:** ME recognition performance of OC-Net.

Database	Accuracy (%)	F1-Score (%)
Overall	78.53	67.80
CASME II	90.80	86.21
SAMM	69.19	53.79
SMIC	75.20	62.80

**Table 7:** Performance comparison of OC-Net variants.

Networks	Accuracy (%)	F1-Score (%)
OC-Net without dropout	76.87	65.31
OC-Net with dropout in 1st layer	77.17	65.76
OC-Net	78.53	67.80
OC-Net with dropout in 1st, 2nd and 3rd layers	78.23	67.35

**Table 8:** OC-Net with data augmentation for ME analysis.

Database	Accuracy (%)	F1-Score (%)
Overall	79.14	68.71
CASME II	90.80	86.21
SAMM	71.72	57.58
SMIC	74.80	62.20

## Data Availability

The dataset can be downloaded from http://fu.psych.ac.cn/CASME/casme2-en.php.

## Abbreviations

The following abbreviations are used in this manuscript:

ME	Micro-Expression
CNN	Convolutional Neural Network
VGG-M	Visual Geometry Group Model
SW-Max	Sliding window of maximum label intensity
Max	Maximum Frame
GAP	Global Average Pooling
